# Gastroscopic observation and dose-volume histogram parameter study on gastrointestinal mucous injury for pancreatic cancer treated with TOMO

**DOI:** 10.1097/MD.0000000000038469

**Published:** 2024-06-21

**Authors:** Hualin Wei, Wei Han, Xianbo Zhang, Ming Zhang

**Affiliations:** a Department of Oncology, Hebei General Hospital, Shijiazhuang 050051, Hebei, PR China.

**Keywords:** duodenum, gastroscope, pancreatic cancer, predictor, radiation injury, radiotherapy, stomach

## Abstract

To explore the relationships between gastrointestinal radiation injuries of pancreatic cancer patients treated with TOMO and dose-volume histogram parameters prospectively. Seventy patients with pancreatic cancer who underwent TOMO were enrolled in this prospective study from February 2015 to May 2020. The clinical and dose-volume histogram parameters of the patients were collected. The optimal dose parameters for gastrointestinal radiation ulcers were confirmed based on the receiver operating characteristic curve (ROC) and the area below the ROC curve. Acute gastrointestinal tract toxic and side effect and injury grading correlation analyzed by Kruskal-Wallis rank sum test. Gastrointestinal injury often occurs during radiotherapy for pancreatic cancer, as observed using gastroscopy. The main adverse reactions were radioactive gastrointestinal inflammation (58.5%), radioactive gastrointestinal ulcers (41.4%), active bleeding (10%), newly-developed gastric retention (8.6%), and gastric varices (5.7%). As for the stomach, Dmean and V10 were related to radiation ulcer injury. ROC curve indicated that for stomach a Dmean of 13.39 Gy (area under ROC curves = 0.74, *P* = .048) and a V10 of 72.21% (area = 0.74, *P* = .048) was the tolerated dose for the injury of stomach radiation ulcer. As for duodenum, aV20 and aV25 are related to radiation ulcer injury. ROC curve indicated that aV20 of 22.82 cm^3^ (area = 0.68, *P* = .025) and aV25 of 32.04 cm^3^ (area = 0.66, *P* < .047) was the tolerated dose for the injury of duodenum radiation ulcer. The acute gastrointestinal tract toxic and side effects have no significant correlation with injury grading under gastroscope. Dmean > 13.39 Gy and V10 > 72.21% were the key dosimetric indices for predicting radiation-induced gastric ulcer, and aV20 > 22.82 cm^3^ and aV25 > 32.04 cm^3^ were for duodenal. Gastrointestinal reactions cannot be used as an overall basis for the diagnosis of gastrointestinal injury, and gastroscopy is recommended as a review item after radiotherapy.

## 1. Introduction

Pancreatic cancer is a highly malignant tumor with a poor quality of life and a short survival time.^[1]^ Due to the insidious onset of pancreatic cancer, peripancreatic lymph node metastasis, and easy invasion of large blood vessels, about 80% of the cases are inoperable when found,^[2]^ 25% to 62% cases fail to undergo chemotherapy.^[3–5]^ However, radiotherapy plays an increasingly important role in the treatment of pancreatic cancer in recent years.^[6]^ Currently, the direction for radiotherapy improvement includes the escalation of radiation dose through conventional fractionation,^[7,8]^ stereotactic body radiotherapy,^[9]^ intraoperative radiation etc; unfortunately, as side effects, the neighboring organs like stomach and duodenum will be exposed to some degree of irradiation, causing radiation injuries in gastrointestinal mucosa,^[10]^ thus greatly undermining the security and local control of radiotherapy. Therefore, it is very necessary to study the gastrointestinal radiation injuries and their affecting factors dose parameters and clinical factors etc, according to the recent clinical reports, are related to gastrointestinal toxicity of pancreatic cancer treated after radiotherapy.^[11]^ As for other tumors, such as lung cancer, the parameters of dose-volume histogram (DVH) can be used to predict the toxicity of normal tissues^[12]^; there are also available data to quantify the risks of gastrointestinal toxicity for pancreatic cancer treated with radiotherapy,^[13,14]^ but gastrointestinal toxicity was not evaluated by gastroscopy. In this study, we carried out gastroscopy for pancreatic cancer patients before and after radiotherapy with an aim to describe gastrointestinal mucosa injury on an objective basis and explore whether the gastrointestinal DVH parameters can be used to accurately predict gastrointestinal mucosa injury, thus to provide dosage reference to prevent gastrointestinal injury for pancreatic cancer patients treated after radiotherapy.

## 2. Materials and methods

### 2.1. Patient selection

Seventy patients with pancreatic cancer who received TOMO radiotherapy in the Department of Radiation Oncology from February 2015 to May 2020 were selected. The inclusion criteria were as follows: age ≤ 85 years, KPS > 60; diagnosed as pancreatic cancer patients by histopathology, iconography (CT/MRI/PET-CT) and laboratory examination; no operation indication found by surgical clinic, or being unable to receive operation due to internal diseases or patient refusing operation or patients only undergoing exploratory laparotomy, clinical stages confirmed at I to IV. The exclusion criteria were as follows: stomach or duodenum being surgically removed; no gastroscope records before or after radiotherapy; stomach or duodenum being far from PTV, and stomach or duodenum not delineated in radiotherapy program.

### 2.2. Radiotherapy

The radiotherapy technology applied is TOMO with Helical Tomotherapy. Tumors of all selected patients were positioned with Siemens 16-Slice 75 cm Aperture Spiral CT scanning. Before positioning, contrast medium was taken orally, and the scanning area was 2 cm above dome diaphragm till the lower pole of the kidney with interlayer spacing 4 mm. The gross tumor volume (GTV) was delineated according to the CT scan imagery and PET/CT, the 5 mm outreach of GTV is defined as clinical target volume (CTV), and the 5 mm even-outreach of CTV defined as PTV, GTV is the GTV and lymphatic metastasis with reference to imaging data. Dose scheme: GTV/CTV/50-80 Gy/40-65 Gy/10-25f, 1f/d, 5f/w; the prescribed dose shall cover at least 95% of the target volume.

### 2.3. Dose parameters

The dose parameters of DVH are chosen as follows: Gastroduodenal Dmax, Dmin, Dmedian, Dmean, D3mL, D5mL, D10mL, D15mL (the irradiated dose to 3, 5, 10, and 15 mL of volume), Vdose: the percentage of volume receiving more than the irradiated dose and a Vdose: the percentage of absolute volume receiving more than the irradiated dose.

### 2.4. Gastroscope assessment

Before the radiotherapy, all the patients (within 1 week before radiotherapy) received gastroscopy; prompt check would follow up, if severe gastrointestinal reaction occurred during the radiotherapy process; when radiotherapy was completed, and 1, 3, 6, 9, and 12 months later, gastroscope would be conducted again, the maximum injury grade by the corresponding statistical analysis would be taken as the reference. Radioactive gastrointestinal toxicity is defined as^[15,16]^: radioactive gastrointestinal inflammation: the diffuse erythema, seeping, edema, hyperemia or other types of mucous alteration newly-developed in detected area or the deteriorated cases upon the original lesion; radiation ulcer developed from gastrointestinal inflammation; mucous injury caused by radioactive inflammation or ulcer in detected area which give rise to unexpected active bleeding, thus in need of hemostatic therapy; radiation related gastrointestinal perforation discovered in gastroscopy or by other radiographic means after radiotherapy. All gastroscopic observation was operated by 1 experienced technician, detecting the areas till the horizontal segment of duodenum.

**Figure 1.**
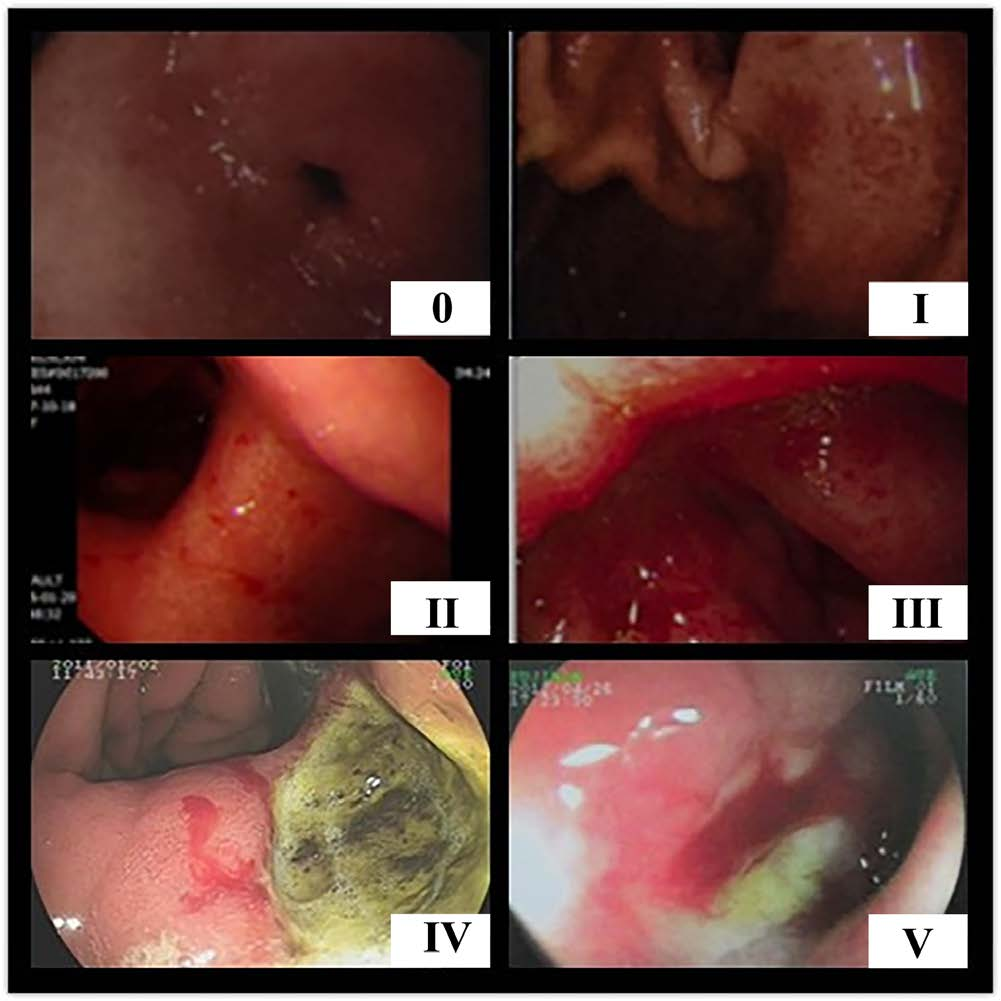
Evaluation standard legend of gastrointestinal mucosal injury after radiotherapy for pancreatic cancer.

According to endoscope observation, gastrointestinal mucous injury evaluation criteria for pancreatic cancer treated with radiotherapy are as follows: G0: smooth mucous membrane, distortion-free, fine villi; G1: mild hyperemia and swelling in mucosa, accompanied by increased fragility in tissue, contact bleeding; G2: petechia or splinter hemorrhage in mucosa; G3: petechia or splinter erosion in mucosa; G4: helcosis in mucosa; G5: mucosal ulceration with bleeding or intestinal strictures (Fig. 1).

### 2.5. Gastrointestinal tract toxic and side effects

According to common terminology criteria for adverse events (CTCAE) Version 5.0, digestive tract toxicity from the beginning of radiotherapy to the first 3 months (acute stage) would be appraised, and the specific time for corresponding symptoms would be recorded.

### 2.6. Statistics

All the DVH parameters are analyzed with receiver operating characteristic (ROC) curve and the area under the ROC, the clinical enumeration data analyzed with chi-square test and measurement data with t-test; the correlation between acute gastrointestinal toxicity and the injury grading under endoscope observation is analyzed with Kruskal-Wallis rank sum test. *P* < .05 was considered statistically significant. All data were analyzed by the SPSS 26.0 statistical software.

## 3. Results

### 3.1. Patients

A total of 70 patients were included in this study. The median age was 59 years (range 33–76 years), and the mean KPS score was 80. There were 42 (60%) males and 28 (40%) females. There were 38 (54.3%) cases of pancreatic head tumors, 19 (27.1%) cases of pancreatic body tumors and 13 (18.6%) cases of pancreatic tail tumors. Twenty-five (36%) cases were confirmed by pathology, and 45 (64%) cases were examined by PET/CT or NMR. Clinical stage: stage I, stage II, stage III and stage IV, accounting for 1.4%, 7.1%, 55.7%, and 35.7%, respectively. Among them, 17 (24.3%) patients underwent surgery to reduce jaundice. Fifteen (23%) cases had gastric and duodenal tumor invasion. There were 14 (20%) cases with diabetes history, 6 (8.6%) cases with ulcer history, 26 (37.1%) cases with HP infection history, 21 (30%) cases with smoking history, and 22 (31.4%) cases with drinking history.

### 3.2. Endoscopic performance

**Figure 2.**
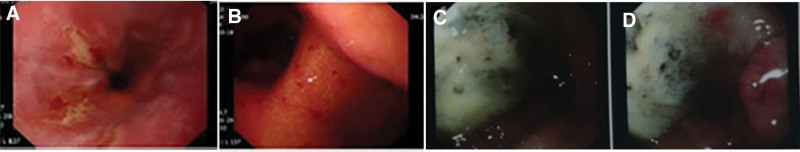
Endoscopic performance. (A, B) general gastrointestinal inflammatory changes after radiotherapy. (C, D) severe gastrointestinal changes after radiotherapy.

All patients were given gastroscopy before radiotherapy, and the following problems were found: 11 patients were detected with dysfunction (1 case of gastric retention, 2 cases of reflux esophagitis, 4 cases of gastric varices and 4 cases of duodenal bulb ulcer). The median time of gastroscopy after radiotherapy is 1 month (range 0–22 months). Gastroscopic performance after radiotherapy is shown as follows (Table 1): radiation gastritis 15 (21.4%) cases, radiation duodenitis 11 (15.7%) cases, inflammation in both stomach and duodenum 15 (21.4%) cases, total inflammation 41 (58.5%) cases; gastroscopic performance can be concluded as mucosal hyperemia, edema, erosion, accompanied by increased fragility in tissue, contact bleeding spots, and diffuse lesion in general (Fig. 2A and B). Radiation gastric ulcers 11 (15.7%) cases, radiation duodenal ulcers 17 (24.2%) cases, ulcers in both stomach and duodenum 1 (1.4%) case, total ulcers 29 (41.4%) cases. Among them, 25 (35.7%) cases were newly-developed ulcers after radiotherapy, 4 (5.7%) cases were aggravated from the previous duodenal bulb ulcers. Individual or multiple shallow or deep ulcers were detected with gastroscope, with white fur on surface; the severe cases were accompanied with bleeding or intestinal stricture (Fig. 2C and D). Among them, 7 (10%) cases were active bleeding incurred by radiation were discovered, among which 6 cases were hemorrhage upon ulcer, 1 case of newly-developed active bleeding incurred by gastric varices. There were 3 (4.3%) cases of local stenosis incurred by radiation ulcer and scar, and no ulcer with perforation detected. There were 6 (8.6%) cases of newly-developed gastric retention, 4 (5.7%) cases of gastric varices; the patients with reflux esophagitis or gastric varices had more aggravated symptoms according to gastroscopic results: the original symptoms were still there, and 1 patient with reflux esophagitis was detected with newly-developed gastric-erosion-like inflammation, and 1 case with newly-developed giant ulcers in duodenum; for patients with gastric varices, 1 case was detected with newly-developed radiation gastritis, 2 cases with newly-developed gastroduodenal radiation ulcers and 1 case with gastric ulcer plus hemorrhage.

**Table 1:** Radioactive gastrointestinal toxicity incidence.

Injury types	Case No.	Incidence
Gastritis	15	21.4%
Duodenitis	11	15.7%
Inflammation in both stomach and duodenum	15	21.4%
Gastric ulcer	11	15.7%
Duodenal ulcer	17	24.2%
Ulcers in both stomach and duodenum	1	1.4%

### 3.3. DVH parameter analysis

In order to determine which DVH parameters are related to the gastrointestinal toxicity, we analyzed the radiation ulcers under gastroscope observation as well as the toxicity events such as hemorrhage, stricture and perforation incurred from the ulcers (the G4-G5 events as per endoscopic grading). The dose parameters of ROC curve can be seen in Table 2 and 3. As for stomach, Dmean and V10 are related to the injury of radiation ulcer. When Stomach Dmean > 13.39 Gy, the frequency of ulcer occurrence is 25%, when Stomach Dmean ≤ 13.39 Gy, the frequency dropped to 9.1%, *P* = .048; when stomach V10 > 72.21%, the frequency of ulcer occurrence is 33.3%, when stomach V10 ≤ 72.21%, the frequency of ulcer occurrence 11.8%, *P* = .048. As for duodenum, aV20 and aV25 are related to radiation ulcer injury, when aV20 > 22.82 cm^3^, the frequency of duodenal ulcer occurrence is 39%, when aV20 ≤ 22.82 cm^3^, the frequency of duodenal ulcer occurrence is 10.7%, *P* = .025; when a V25 > 32.04 cm^3^, the frequency of ulcer occurrence is 50%; when aV25 ≤ 32.04 cm^3^, the frequency of ulcer occurrence 19.6%, *P* = .047.

**Table 2:** The stomach part.

Evaluation index	Area under the ROC curve	*P*	cutoff
Stomach D max	0.532	.789	58.85
Stomach D min	0.697	.105	1.41
Stomach D median	0.690	.117	11.56
Stomach D mean	0.740	.048	13.39
Stomach V10	0.740	.048	72.21
Stomach V15	0.727	.062	39.97
Stomach V20	0.706	.091	27.12
Stomach V25	0.675	.149	16.77
Stomach V30	0.693	.113	6.96
Stomach V35	0.684	.130	4.06
Stomach V40	0.667	.171	1.33
Stomach V45	0.606	.383	0.96
Stomach V50	0.606	.383	0.45
Stomach V55	0.667	.171	0.13
Stomach D3mL	0.636	.262	40.43
Stomach D5mL	0.701	.098	38.56
Stomach D10mL	0.732	.057	32.59
Stomach D15mL	0.732	.057	30.67
a Stomach V20 (absolute volume)	0.669	.165	32.38
a Stomach V25	0.675	.149	39.09
a Stomach V30	0.723	.067	16.94
a Stomach V35	0.675	.149	10.40
a Stomach V40	0.636	.262	5.82
a Stomach V45	0.563	.606	3.33
a Stomach V50	0.550	.682	1.22
a Stomach V55	0.600	.413	0.19

ROC = receiver operating characteristic curve.

**Table 3:** The duodenum part.

Evaluation index	Area under the ROC curve	*P*	Cutoff
Duodenum D max	0.576	.335	54.61
Duodenum D min	0.490	.902	1.04
Duodenum D median	0.541	.599	6.68
Duodenum D mean	0.549	.534	23.74
Duodenum V10	0.491	.908	40.99
Duodenum V15	0.475	.753	33.10
Duodenum V20	0.545	.571	32.57
Duodenum V25	0.548	.539	37.05
Duodenum V30	0.530	.702	17.33
Duodenum V35	0.552	.507	10.86
Duodenum V40	0.559	.452	5.69
Duodenum V45	0.565	.408	2.28
Duodenum V50	0.561	.440	0.13
Duodenum V55	0.547	.548	0.02
Duodenum D3mL	0.589	.259	39.89
Duodenum D5mL	0.617	.138	36.42
Duodenum D10mL	0.623	.118	31.47
Duodenum D15mL	0.625	.111	27.57
a Duodenum V20	0.676	.025	22.82
a Duodenum V25	0.656	.047	32.04
a Duodenum V30	0.628	.104	12.33
a Duodenum V35	0.641	.072	6.37
a Duodenum V40	0.607	.172	2.80
a Duodenum V45	0.602	.197	0.66
a Duodenum V50	0.601	.199	0.16
a Duodenum V55	0.594	.232	0.02

ROC = receiver operating characteristic curve.

### 3.4. The correlation of acute gastrointestinal tract toxic and side effects and the injury grading under gastroscope

The occurrence rate of acute gastrointestinal toxic and side effects for I°, II°, III°, IV° were 34.2% (24 cases), 45.7% (32 cases), 18.6% (13 cases), and 1.4% (1 case), respectively. G0-G5 injury grading incidence under endoscope is 28.6% (20 cases), 10% 7 cases), 4.3% (3 cases), 15.7% (11 cases), 28.6% (20 cases), and 12.8% (9 cases), respectively. In order to determine whether the gastrointestinal tract toxic and side effects are related to the injury grading, the Kruskal-Wallis rank sum test was conducted, and the results showed there was no significant correlation between them (*P* > .05) (Table 4).

**Table 4:** The correlation analysis of acute gastrointestinal tract toxic and side effects and the injury grading under gastroscope.

CTCAE	Gastrointestinal toxicity under gastroscope					
	0	1	2	3	4	5
1	11	1	1	4	7	0
2	5	5	2	5	11	4
3	4	1	0	1	2	5
4	0	0	0	0	1	0
*Z*	6.016					
*P*	.111					

CTCAE = common terminology criteria for adverse events.

### 3.5. Second gastroscope comparison

Due to fear or other reasons, most selected patients refused to take gastroscope after radiotherapy and gastroscope rechecks 1, 3, 6, 9, and 12 months after radiotherapy; only 11 patients took 2 gastroscopy observations after radiotherapy. For the 11 patients, the first gastroscope was carried out immediately after radiotherapy; as to the second gastroscope, 6 patients took it 3 months after radiotherapy, 3 took it 6 months later, 1 9 months later and 1 12 months later. By comparison of the 2 gastroscopic checks, radioactive inflammation of 5 patients developed to radiation ulcers, 3 cases’ inflammation or ulcers remained unchanged, 1 case developed gastric retention for the second time, and 1 case remained normal before and after the radiotherapy. Overall, the second gastroscopy caused more severe injury to patients.

## 4. Discussion

Currently, the treatment for advanced pancreatic carcinoma is mostly radiotherapy combined with chemotherapy, and the curative effect is encouraging. However, the symptoms of digestive tract caused by radioactive gastrointestinal toxicity are easily confused with gastrointestinal symptoms of pancreatic cancer. Yet, this disease has not given due attention; The previously reported cases are mainly radiation enteritis, and radioactive gastrointestinal inflammation is rarely reported. In this paper, pancreatic cancer patients received gastroscopy both before and TOMO treatment, with a view to probing into the radioactive gastrointestinal mucous injury. Normally, radiation-induced ulcers develop shortly after the completion of treatment, and the peak time is 1–2 months, the median time we carried out gastroscopy is 1 month, thus to objectively reflect the gastrointestinal injury.

Gastrointestinal tract mucosa injury takes on diversified features: except for the inflammation and ulcers, there are local stenosis, active bleeding from ulcers, gastric retention, gastric varices etc caused by ulcer and scar. This study shows that the total occurrence rate of radioactive inflammation is 58.5%, the total occurrence rate of radiation ulcers is 41.4%, undoubtedly, gastrointestinal toxicity related to radiotherapy is commonplace. Takatori et al^[17]^ used to give gastroscopic observation to 91 pancreatic cancer patients who received proton synchronous gemcitabine chemotherapy, and found that the total occurrence rate of gastrointestinal ulcers related to radiation is 49.4%, very close to the percentage reported in this paper. Yoon et al^[18]^ used to carry out gastroscopy for liver cancer patients who had received radiotherapy 2 months ago and found that the occurrence rate of radioactive stomach inflammation and radioactive duodenum inflammation is 16% and 17%, respectively, while the radioactive stomach ulcer and radioactive duodenum ulcer are 9% and 16% respectively. The injury incidence is a little lower than what is reported in this paper, taking into account the median time we carried out gastroscope is 1 month (the gastroscope time after radiotherapy is yet inconclusive), and the difference in disease type and radiotherapy methods as well as the diverse injury thus incurred, we cannot rule out the possibility that pancreatic cancer radiotherapy is more inclined to cause gastrointestinal injury than that of liver cancer radiotherapy.

The correlation between dose-volume factors and radioactive gastrointestinal toxicity is rarely explored. Gastrointestinal toxicity study for pancreatic cancer patients was mainly aimed at analyzing the duodenum factors with CTCAE as reference, yet factors of stomach are scarce, and there is no established standard for that. Murphy et al^[11]^ used to analyze 73 pancreatic cancer patients who had received SBRT; with CTCAE grade 2–4 gastrointestinal symptoms as reference, he concluded that aV15, aV20 and Dmax were significantly related to gastrointestinal injury. When aV15 ≥ 9.1 cm^3^ and aV15 < 9.1 cm^3^, the duodenum injury occurrence rate is 52% and 11% respectively (*P* = .002); when aV20 ≥ 3.3 cm^3^ and aV20 < 3.3 cm^3^, the injury occurrence rate is 52% and 11% respectively (*P* = .002); when Dmax ≥ 23 Gy and Dmax < 23 Gy, the injury occurrence rate is 49% and 12% respectively (*P* = .04). Different from their results our results were obtained with the more advanced TOMO technology, which can offer more ideal applicability in target area and better dose homogeneity, so the irradiated volume with the same dose could increase, and the security could be better guaranteed. Patrick et al^[19]^ used to do the same study. They analyzed 106 patients with advanced pancreatic cancer who had received 3-D or IMRT chemoradiotherapy; with CTCAE > 2 grade as the injury criteria (including stage 5), they concluded aV55 > 1cm^3^ is related to duodenum injury (*P* = .002). One dose-limit point of high dose they concluded is different from ours, and we did not find any correlation to duodenum aV55. It is noteworthy that they did not analyze the DVH parameters below V40, and their study results were not complete. Jiayi Huang et al^[20]^ used to analyze 46 patients with advanced pancreatic cancer who had received chemoradiotherapy; however, they take CTCAE > 3 grade as criteria, when V25 Gy ≤ 45% and V25 Gy > 45%, the duodenum injury incidence is 8% and 48% respectively (*P* = .03), and when V35 Gy ≤ 20% and V30 Gy > 20%, the injury incidence is 0% and 41% respectively (*P* = .04); at grade 3, the incidence is 37%, 4 times higher than that of Patrick Kelly^[19]^ study results. Obviously, it is not objective if symptoms are taken as injury criteria, and is inclined to lead to differences in evaluation criteria. Our study takes the real radiation ulcers observed under gastroscope as criteria to draw the conclusion that aV20 and av25 are related to duodenum radiation ulcers. In addition, we obtain the dosimetric predictor Dmean and V10 for gastric ulcers of pancreatic cancer patients, and the results are more scientific and objective. Currently, the study of Yoon et al^[18]^ on liver cancer is the most similar to ours, in which the 90 patients analyzed also received gastroscopy before and after radiotherapy, and CTCAE is graded with injury degree according to gastroscopic observation; for stomach, V25 Gy is an important predictor for gastric injury ≥ 2 grade, when V25 Gy ≤ 6.3% and V25 Gy > 6.3%, the incidence of injury is 2.9% and 57.1% respectively. With regard to duodenum, V35 Gy is an important predictor for duodenum injury ≥ 2 grade, when V35 Gy ≤ 5.4% and V35 Gy > 5.4%, the incidence of injury is 9.4% and 45.9% respectively. Most of the selected patients in their study suffered from liver cirrhosis, and the risk of upper digestive tract bleeding is far higher than that of our patients, and the interference effect on dose factors from portal hypertension incurred by liver cirrhosis cannot be excluded. Since there is enterogastric peristalsis, stomach and duodenum cannot be well fixed in targeted area, and there will be a certain deviation if dose-volume is applied to evaluate the danger dose; therefore, there is no established conclusion to determine the proportion of dosage against the gastrointestinal volume in developing radiotherapy plan. Our study results can serve as an important dosage reference for TOMO treatment, with which the radioactive ulcer could be effectively prevented and the life quality of pancreatic cancer patients could be improved.

The pancreatic cancer patients are from different stages of disease, and there are multiple and complex clinical factors. Currently, the radioactive gastrointestinal study has not proved the correlation between gastrointestinal injury and the age, sex, smoking, alcohol intake, KPS grading etc, but some dangerous anamnesis are reported to be significantly relevant, for example, Bae et al^[21]^ used to analyze 6 cases of severe gastrointestinal toxicity after intraperitoneal tumor radiotherapy, and pointed that history of ulcer could be taken as a clinically significant predictor before radiotherapy, and Papatheodoridis et al^[22]^ reported that HP infection was significantly related to the gastrointestinal mucous injury.

Radioactive gastrointestinal reaction is divided into acute stage and advanced stage; the acute stage (within 3 months of radiotherapy) is followed by damage of mucosal cells, the mucosa hyperemia and edema of various degrees, as well as increased fragility of tissue; under gastroscope, it shows patchy bleeding and edema in gastrointestinal mucosa while in clinical manifestation, it shows nausea, vomiting and abdominal pain. The advanced stage (3–6 months after radiotherapy or even longer) is followed with vascular lesions under mucosa, endarteritis obliterans and endotheliosis, thus leading to ischemia ulcer of mucosa, interstitial fibrosis; under gastroscope, it mainly shows deep and shallow ulcers as well as stricture and distortion. In clinical manifestation, it shows pain under xiphoid, anorexia, nausea, vomiting and hematochezia; for serious cases, repeated hematochezia and hematemesis are reported as symptoms. Eleven of our patients received a second gastroscopy after radiotherapy; by comparing the results, we found the lesions of most patients were worsening after the second gastroscopy, conforming to the injury characteristics of acute stage and advanced stage; it also indicates there tend to be hysteresis in radiation injury: when patients have no obvious gastrointestinal tract symptom, it does not necessarily mean there is no injury in later period after radiotherapy, so it is advisable for patients to receive regular gastroscopy after radiotherapy and symptomatic treatment on a proactive basis; only in this way, further possible troubles could be avoided.

During the radiotherapy period, we also observed the patients’ acute gastrointestinal tract reaction; for cases of the acute stage, the median time of reaction is 7 days after radiotherapy, with symptoms such as anorexia, nausea, vomiting, dyspepsia and constipation etc, the symptoms were graded as per CTCAE criteria. With the Kruskal-Wallis rank sum test, digestive tract toxicity grading and the injury grading under gastroscope are proved to be irrelevant (*P* = .111). It can be seen that the gastrointestinal reaction in acute stage during radiotherapy is barely related to the injury degree observed under gastroscope in later period, and the clinical symptoms of gastrointestinal mucous injury are nonspecific, inclined to be mistaken as the correlated symptoms of tumors themselves; moreover, the light gastrointestinal reaction does not necessarily mean there will not be ulcer injury in later period. Gastrointestinal reaction cannot be used as the basis for the diagnosis of gastrointestinal injury. Gastroscopy must be performed regularly to determine whether there is injury and the type of injury.

Our study has the following advantages: First, we chose patients with high risks, the stomach and duodenum of whom are close to PTV in target delineation. Second, all the gastrointestinal toxicity cases in our study have been observed under endoscope, and radiation ulcer under endoscope have been taken as criteria; by contrast, in the previous report, gastroscopy has often been carried out among patients with abdominal symptoms, thus selection bias is more likely to occur. Third, all the patients in our study have been treated with the advanced TOMO technology. Yet this study is not without limitations: For one thing, 25 patients have been excluded, of whom 11 lacked gastroscopy results, 8 refused to receive gastroscopy after radiotherapy, so the radioactive ulcer occurrence might be lower than estimate; for further study, we have to pay more attention to coordinating work, and try our best to avoid selection bias due to the lack of gastroscopy. For another, of the selected 70 patients, 45 are not yet proved with pancreatic cancer pathologically, and moreover, our results are based on the endoscope observation of complicating diseases in upper digestive tracts after TOMO treatment, and the symptoms analyzed are mainly acute phase reaction; the median time of endoscope check is 1 month, but honestly, the injury itself is evolutive: the inflammation may get healed or deteriorated to ulcers; the injury blowout period after radiotherapy is not confirmed. For further study, gastroscopy should be increased in frequency and carried out for patients 3, 6, 9, months, respectively, after radiotherapy; the comparison of injury incidence and detection of injury burst time are particularly important.

## 5. Conclusions

After radiotherapy for pancreatic cancer patients, radioactive gastrointestinal inflammation and ulcer are commonplace, and gastroscopy is necessary after radiotherapy. DVH parameters are closely related to radiation injury, so stomach D mean > 13.39 Gy, stomach V10 > 72.21%, duodenum aV20 > 22.82 cm^3^ and duodenum aV25 > 32.04 cm^3^ can be used as reference to develop plans for pancreatic cancer treated with TOMO; In addition, protective treatment should be emphasized in early stages thus to reduce the risk of gastrointestinal injury after radiotherapy for pancreatic cancer patients. Gastrointestinal reaction cannot be taken as the overall basis for diagnosing gastrointestinal injury, and regular gastroscope check is required to confirm whether there are gastrointestinal mucous injury as well as the types of injury.

## Author contributions

**Conceptualization:** Ming Zhang.

**Data curation:** Hualin Wei, Ming Zhang.

**Formal analysis:** Hualin Wei.

**Software:** Wei Han.

**Validation:** Wei Han, Xianbo Zhang.

**Visualization:** Xianbo Zhang.

**Writing – original draft:** Hualin Wei.

**Writing – review & editing:** Ming Zhang.
